# A review of the potential for competitive cereal cultivars as a tool in integrated weed management

**DOI:** 10.1111/wre.12137

**Published:** 2015-01-26

**Authors:** I K S Andrew, J Storkey, D L Sparkes

**Affiliations:** ^1^ Department of AgroecologyRothamsted ResearchHarpendenHertfordshireUK; ^2^ University of NottinghamSutton BoningtonLeicestershireUK; ^3^ WURthe Netherlands

**Keywords:** cultural weed control, plant functional traits, suppression, tolerance

## Abstract

Competitive crop cultivars offer a potentially cheap option to include in integrated weed management strategies (IWM). Although cultivars with high competitive potential have been identified amongst cereal crops, competitiveness has not traditionally been considered a priority for breeding or farmer cultivar choice. The challenge of managing herbicide‐resistant weed populations has, however, renewed interest in cultural weed control options, including competitive cultivars. We evaluated the current understanding of the traits that explain variability in competitive ability between cultivars, the relationship between suppression of weed neighbours and tolerance of their presence and the existence of trade‐offs between competitive ability and yield in weed‐free scenarios. A large number of relationships between competitive ability and plant traits have been reported in the literature, including plant height, speed of development, canopy architecture and partitioning of resources. There is uncertainty over the relationship between suppressive ability and tolerance, although tolerance is a less stable trait over seasons and locations. To realise the potential of competitive crop cultivars as a tool in IWM, a quick and simple‐to‐use protocol for assessing the competitive potential of new cultivars is required; it is likely that this will not be based on a single trait, but will need to capture the combined effect of multiple traits. A way needs to be found to make this information accessible to farmers, so that competitive cultivars can be better integrated into their weed control programmes.

## Introduction

In an agricultural system, the aim is to produce the highest yield achievable whilst minimising costs. In comparison with pests and diseases, weeds have the potential to incur the greatest yield loss, through competition with the crop and decreasing yield quality, and can, therefore, incur high costs of control (Oerke, 2006). However, the introduction of herbicides has allowed very effective and relatively cheap control of weed species, relieving arable farmers of a heavy financial burden and contributing to the increase in average yield seen during the period of their adoption (Moss *et al*., 2004). Unfortunately, the over‐reliance on chemical control has also led to a number of environmental and agronomic concerns. The application of herbicides, combined with other changes in farmland management, is leading to the reduction of non‐weedy species and having impacts on farmland biodiversity and ecosystem function (Moonen & Bàrberi, 2008; Storkey *et al*., 2012). More importantly, from a production perspective, herbicide resistance is now widespread amongst many problematic weed species, encouraged by the increasing dependence upon a limited number of active ingredients (Heap, 1997; Moss *et al*., 2011). In the UK, herbicide‐resistant *Alopecurus myosuroides* Huds. can be found on at least 16 000 farms in 34 counties (Moss *et al*., 2011). In addition, EU regulations are reducing the number of herbicide options, and new modes of action are proving elusive, further increasing the risk of the development of resistance to the remaining products (Duke, 2012). In response to these challenges, there is renewed interest in the potential for integrating non‐chemical (or ‘cultural’) control options into weed control strategies.

Many cultural control methods may be employed by farmers to reduce weed populations, including delayed drilling, increased seed rate and rotational ploughing (Lutman *et al*., 2013). Competitive cultivars are a potentially attractive option in comparison, because they do not incur any additional costs. Such cultivars may be more capable of reducing the fitness of a weed species through competition for limited resources (Christensen, 1995), may produce chemical exudates that reduce growth (Wu *et al*., 1999) and reduce the economic burden of weeds by resisting yield loss (Reeves & Brooke, 1977; Vandeleur & Gill, 2004). Competitive cultivars could reduce the seed return of a weed species and contribute to medium to long‐term weed management strategies, reducing the pressure on herbicides and improving the sustainability of cropping systems. For example, in Greece, the use of competitive cultivars alone has already been demonstrated to allow for a 50% reduction in recommended levels of herbicides in wheat (Travlos, 2012).

Variability between cereal cultivars in their ability to resist yield losses from weed competition was demonstrated in the 1970s. Four winter wheat cultivars were compared for their ability to withstand competitive pressure, with a range of 28–39% reduction in yield when *Lolium multiflorum* L. was present at *c*. 100 plants m^−2^ (Appleby *et al*., 1976). Differences between cultivars were also shown between 29 cultivars in competition with *Lolium rigidum* Gaudin*,* where, at densities of 1500 weeds m^−2^, yield reductions ranged from 23.1% to 47.8% (Reeves & Brooke, 1977). As modern, short‐strawed cultivars entered the market, variation in their capacity to decrease weed fitness continued to generate research interest. For example, two cultivars of wheat and barley showed different capacities to reduce the number of seed heads produced by *A. myosuroides* (Moss, 1985). Wicks *et al*. (1986) demonstrated that winter wheat cultivars differed in their ability to negatively impact on the establishment and subsequent growth of the summer annuals *Amaranthus retroflexus* L., *Portulaca oleracea* L., *Echinocholoa crus‐galli* (L.) Beauv. and *Eragrostis cilianensis* (All.) Vign. ex Janchen. In this study, control of weeds ranged from 59% to 96% when compared to plots where the crop had been removed by cultivation prior to May.

Comparative studies of this type are, however, of limited value outside of the experimental pool of cultivars. It is important that more predictive approaches are developed that can be used to assess new cultivars or guide future crop breeding efforts. With this in mind, this review discusses first the distinction between suppression and tolerance and then assesses what is known about the suite of traits that determines how competitive ability varies between cultivars. We focus primarily on the cereals wheat (*Triticum aestivum* L.), barley (*Hordeum vulgare* L.) and oats (*Avena sativa* L.). Allelopathy has more recently started to attract interest in cereals and has been demonstrated to be of importance in both wheat and barley in determining competitive outcomes (Bertholdsson, 2005), but is not considered here in detail. For a recent review of allelopathy in cereal crops, see Worthington and Reberg‐Horton (2013).

## Suppression versus tolerance of weeds

Two aspects of cultivar competitiveness can be defined. The first is the ability of the crop to reduce the fitness of a competitor, and the second is the ability of the crop to withstand the competitive impact of neighbours and resist yield loss (Goldberg, 1990; Grace, 1990). These are referred to by different terms in the literature, but here will be described, respectively, as ‘suppressive ability’ and ‘tolerance ability’ (Hansen *et al*., 2008). There is value in defining suppression and tolerance, as both have different outcomes in terms of weed management. In the presence of a strong suppressive cultivar, weed species will have reduced seed production, which is a viable part of a long‐term strategy in weed control. By contrast, tolerance means yield will be maintained under weed pressure (Kirkland & Hunter, 1991; Cosser *et al*., 1997). However, it does not necessarily imply any control is exerted on a weed population, potentially allowing it to reach levels that can no longer be tolerated (Appleby *et al*., 1976; Jordan, 1993; Lemerle *et al*., 1996).

Variation in suppressive ability between cultivars of winter wheat and spring barley have been observed against volunteer oil seed rape (*Brassica napus* L.) (Christensen *et al*., 1994; Christensen, 1995). Similar results have been observed in wheat against *L. rigidum* (Lemerle *et al*., 1996), *Aegilops cylindrica* Host (Ogg & Seefeldt, 1999), *Galium aparine* L. (Mennan & Zandstra, 2005) and weed mixtures (Wicks *et al*., 1986; Cosser *et al*., 1997; Korres & Froud‐Williams, 2002). Differences in tolerance ability have been identified between cultivars of wheat (Appleby *et al*., 1976; Reeves & Brooke, 1977; Challaiah *et al*., 1986; Vandeleur & Gill, 2004; Zerner *et al*., 2008) and barley (O'Donovan *et al*., 2000). Tolerance has been observed to vary little between winter wheat cultivars in comparison with suppressive ability (Jordan, 1993; Olesen *et al*., 2004). Additionally, tolerance is often reported as being inconsistent over seasons and locations (Cousens & Mokhtari, 1998; Olesen *et al*., 2004). This has led to the suggestion that competitive tolerance does not exist as a mechanism to resist the suppressive effects of a neighbour sharing finite resources. So‐called tolerance traits may instead be stress‐resistance traits (Wang *et al*., 2010).

As the distinction between suppression and tolerance has been defined, research has sought to establish if they are correlated. Traits have typically been defined as having either suppressive or tolerance qualities (Huel & Hucl, 1996; Coleman *et al*., 2001) and, although some have been suggested to confer both (such as the assessment of wheat height in Seefeldt *et al*. (1999), there is no clear consensus in the literature on how they are linked. Negative relationships between tolerance and suppressive ability have been reported; (Miller & Werner, 1987), positive relationships in other cases (Goldberg & Fleetwood, 1987; Watson *et al*., 2006) or no perceivable relationship at all (Goldberg & Landa, 1991; Cahill *et al*., 2005). We contend that suppressive ability and tolerance are best considered as separate entities, due to the uncertainty of the relationship between them and, where possible, we maintain this distinction throughout. To describe a trait broadly as conferring a ‘competitive advantage’ could be misleading, as it may suggest that the trait confers both suppressive and tolerance ability (Lemerle *et al*., 2001; Cahill *et al*., 2005). Although, in this review, we consider both tolerance and suppression, the emphasis is on weed suppression, as this is more important in the context of integrating competitive cultivars into an IWM strategy.

## The role of traits in competitive ability

The term ‘trait’ is used in ecology for a characteristic which may be used as a predictor of fitness in different environments. Some confusion has surrounded the use of this term. There are attempts to split definitions into levels of organisation, with the term ‘trait’ being reserved for any feature that is morphological, physiological or phenological and can be identified and measured at the level of the individual (Violle *et al*., 2007). This literature review will adhere to this definition, although target traits in some articles do not match the criteria and, indeed, competitive ability itself is often referred to as a trait.

### Height

Early interest in competitive cultivar traits mainly focussed on maximum canopy height. This originated from the observed differences between the ‘new’ semidwarf and ‘old’ (and often taller) cultivars of wheat. Whilst lower yielding in weed‐free situations, taller cultivars were typically better tolerators of weed pressure and suppressors of weed growth (Appleby *et al*., 1976; Challaiah *et al*., 1986; Lemerle *et al*., 1996; Ogg & Seefeldt, 1999; Vandeleur & Gill, 2004). The benefit of height has been demonstrated in wheat in competition with *Bromus tectorum* L. (Challaiah *et al*., 1986), *B. napus*‐infested spring barley (Christensen, 1995), winter wheat against *A. cylindrica* (Ogg & Seefeldt, 1999) and oats, barley and wheat against *G. aparine* (Brain *et al*., 1999).

Although the advantages of plant height in terms of shading weeds are clear, it cannot, alone, explain variation in competitive ability. Wicks *et al*. (2004) compared thirteen red winter wheat cultivars in their ability to suppress a mixture of annual weeds. Their selection covered a broad spectrum of mature heights and discovered a negative correlation between total annual weed density and mature winter wheat height. However, two of the shortest cultivars exhibited stronger suppressive abilities than many tall cultivars. This was an indication that competitive ability cannot be attributed to a single trait, as has since been acknowledged by many authors (Moss, 1985; Lemerle *et al*., 1996; Roberts *et al*., 2001; Mennan & Zandstra, 2005; Watson *et al*., 2006). The relative contribution of height to suppression and tolerance has often been linked to the ability to intercept photosynthetically active radiation (PAR) (Wicks *et al*., 1986; Gooding *et al*., 1993; Lemerle *et al*., 1996), but a strong relationship between heights and PAR interception has not always been found (Blackshaw, 1994). The relative importance of height may be also related to weed species. *Veronica hederifolia* L., a shade‐tolerant species, achieved highest biomass under the tall cv. Maris Widgeon compared with shorter semidwarf cultivars, and the authors suggested that the species benefited from shade during establishment (Gooding *et al*., 1993). A range of other traits, reviewed below, have also been associated with weed suppression or tolerance in cereal cultivars.

### Early vigour

Certain plant strategies are more successful than others under particular environmental pressures (Grime, 1977, 1979). The arable farming environment selects for ruderal traits, including rapid emergence (Didon, 2002) and high biomass accumulation early in the establishment phase (Grime, 2001), both adaptations to fertile environments with high disturbance. Early vigour of a cultivar is related to crop establishment and the rate at which aboveground material is produced and has been correlated with morphological leaf traits such as leaf area in the earliest phases of growth (Rebetzke & Richards, 1999). Traits relating to leaf size, specific leaf area and rate of production vary between cultivars and have been linked to higher suppressive ability (Huel & Hucl, 1996; Coleman *et al*., 2001; Rebetzke *et al*., 2004; Vandeleur & Gill, 2004; Zerner *et al*., 2008). Leaf traits at the early stages of growth have a high heritability, suggesting that early vigour may be selected for in breeding programmes (Rebetzke & Richards, 1999; Coleman *et al*., 2001).

Other indicator traits of early vigour, including early crop height and cover, are potentially useful for assessing variation between cultivars in suppressive ability (Olesen *et al*., 2004; Worthington *et al*., 2013) and could be valuable for designing screening protocols (see below). Bertholdsson (2005, 2011) used early crop mass as an indicator of vigour in wheat and barley and found it to be one of two traits (along with allelopathy) that significantly contributed to suppression of *Lolium perenne* L. and volunteer *B. napus* (oilseed rape) across all years of study. Bread wheat and durum wheat (*Triticum durum*) cultivars that were more competitive against *L. rigidum* also had high vigour, acquiring higher biomass at the seedling stage (Lemerle *et al*., 1996).

The importance of early growth traits in determining variance in competitive ability can be understood in the context of size‐asymmetric competition, as larger plants have a greater capacity to acquire resources than a smaller neighbour and the competitive advantage therefore becomes progressively greater through the season (Weiner & Thomas, 1986). This has also been demonstrated theoretically from a sensitivity analysis of an ecophysiological model of crop/weed competition that found the early growth rate before competition for resources began was the most important parameter in determining the outcome of competition (Kropff *et al*., 1992).

Christensen (1995) found that faster developing cultivars of spring barley were better suppressors of weeds. The importance of early height over mature height was demonstrated by Ogg and Seefeldt (1999), whose most tolerant and suppressive cultivars in the presence of *A*. *cylindrica* were those that increased height at a faster rate. These were the tallest cultivars during growth (but not necessarily at maturity), and their competitive strength was partially attributed to corresponding root growth and the increased water uptake early in the season. Other studies have also found that wheat cultivars with late spike emergence were less tolerant of weed competition and suffered greater yield loss when grown with weeds (Huel & Hucl, 1996; Mason *et al*., 2008). These findings may support the ‘received wisdom’ that cereal cultivars which initiate upwards growth earlier have a competitive advantage, but there would be value in further understanding the processes driving crop/weed interactions at this key developmental stage.

### Tillering

Crop tillering as a trait in competition is commonly measured in three different ways – rate of tiller production, final tiller number and tiller economy (% of tillers surviving). Rate of tiller production and final tiller number are morphologically plastic and density dependent; tiller numbers reduced with increased inter‐ and intraspecific competition. This has been demonstrated in wheat, barley and oats and can vary between cultivars (Huel & Hucl, 1996; Seavers & Wright, 1997; Champion *et al*., 1998). This translates to fertile head production and, consequently, yield reduction in weedy scenarios (Kirkland & Hunter, 1991; Satorre & Snaydon, 1992). Therefore, if tiller loss is not taken into consideration, tiller counts and rate of tillering from individual plants as a trait in crop–weed interactions may give misleading results.

The difficulty of separating density‐dependent effects from the innate capacity of cultivars to produce and maintain tillers may explain the lack of agreement between studies regarding the contribution of tiller number to competitive ability. Tillering capacity in wheat contributed to suppression of dry matter production in mixed flora assemblages (Korres & Froud‐Williams, 2002). Challaiah *et al*. (1986) confirmed the negative relationship between tiller number and seed production of *B. tectorum,* but this was not consistent across sites. Higher tiller numbers also reduced seed production of *L. rigidum* in Australia (Lemerle *et al*., 1996). Other work indicates that tiller number has little or no value in suppressing weeds (Moss, 1985; Wicks *et al*., 1986; Champion *et al*., 1998; Didon & Bostrom, 2003). It may be that the benefit of greater tiller number will be most evident at low crop densities, as, in such situations, they increase the shading ability of the crop stand (Hoad *et al*., 2006) – see section, below, on integrating competitive cultivar traits with other cultural control options. Tiller economy in weedy situations would benefit from further study. This may be considered, in itself, not only a tolerance trait (as it indicates maintaining yield under competitive pressure), but also a suppressive trait due to a cultivar's ability to maintain high levels of light interception (Challaiah *et al*., 1986). This was demonstrated by Seavers and Wright (1997), in a study of cultivars of wheat, barley and oats, where cultivars with greater tiller economy were those with a superior suppressive ability.

### Canopy architecture

As opposed to focussing on individual traits, such as tiller number or seedling growth rate, other authors have considered a broader measure of ‘canopy architecture’ to be useful for determining variance between cultivars in competitiveness (Davies *et al*., 2004; Hoad *et al*., 2006). Violle *et al*. (2007) would possibly define canopy architecture as a performance trait – the conglomerate influence of many ecophysiological traits, which directly or indirectly influences individual fitness. A number of previous sections have covered facets of canopy architecture, but broad measures of canopy architecture deserve consideration because of their potential usefulness in the development of screening protocols for new varieties.

Various facets of canopy architecture have been measured using a range of methods that can be difficult to reconcile. In wheat and barley, leaf area index at early growth phases was associated with suppression (Huel & Hucl, 1996; Hoad *et al*., 2006; Hansen *et al*., 2008). Coleman *et al*. (2001) quantified various potential measures of canopy architecture and concluded that canopy height, width and length of leaf 2, tiller number and size of flag leaf all contributed to suppressive ability, but relative importance differed between the 2 years of study. Seavers and Wright (1997) noted the importance of leaf size, combined with canopy height and tillering, in a study of wheat, barley and oats in competition with *G. aparine* and compared the growth form of two wheat cultivars. The more erect cultivar with upright leaves was less suppressive than the cultivar with larger, less rigid leaves, but only in one of the 2 years of study. However, Paynter and Hills (2009) could not explain differences in barley competitive ability against *L. rigidium* with growth habit or associated traits, such as plant height and tiller number.

In some cases, a PAR meter has been used to quantify the level of light penetration, capturing the combined effect of these variables. Such devices confirm that the most suppressive cultivars are those that intercept the most PAR (Didon & Hansson, 2002). Taller cultivars do not always transmit the least PAR through their canopies (Blackshaw, 1994), but often height does relate to PAR interception (Gooding *et al*., 1993; Champion *et al*., 1998). Measuring PAR may present a simple way to assess the suppressive ability of a cultivar. However, increased shading is also the primary mechanism by which increased seed rate suppresses weeds, as it influences canopy structure at a population level, thus any studies on differences between cultivars in canopy architecture must take into account seed rate and other factors such as row width.

Cereal leaves differ in their arrangement during growth (Davies *et al*., 2004). The importance of structure at different growth stages is significant when its interaction with cultivar is considered (Hoad *et al*., 2008). Cultivars that are planophile at the early growth stages have been shown to be more suppressive (Huel & Hucl, 1996; Hoad *et al*., 2006). Challaiah *et al*. (1986) measured wheat canopy diameter in early June and found that, when coupled with height, it provided a good means to predict competitive outcomes. Leaf angle in spring barley at growth stage 65 was indicative of suppressive ability (Hansen *et al*., 2008). Changes in canopy architecture through the season also need to be interpreted in the context of the growth habit of different weed species. How canopy architecture contributes to tolerance abilities is less clear. High leaf area index indicates higher tolerance in some studies (Zerner *et al*., 2008), but not others (Huel & Hucl, 1996).

### Belowground traits

In comparison with aboveground canopy measurements, belowground traits have received relatively little attention in cereal crop–weed interactions. This is partly due to the difficulties associated with measuring root traits, particularly when incorporating them into a screening protocol for new cultivars. However, studies have indicated that root competition can be stronger than competition for light, particularly for nitrogen (Exley & Snaydon, 1992; Satorre & Snaydon, 1992; Lucas Bueno & Froud‐Williams, 1994; Stone *et al*., 1998; Lamb *et al*., 2007). Studies on other species have suggested that traits such as root length density, root elongation rate, number of root tips and total root length are determinants of competitive outcomes (Fargione & Tilman, 2006; Stevanato *et al*., 2011). At this stage, little variation in root traits between cereal cultivars has been quantified, implying a lack of opportunity to select cultivars for competitive ability (Satorre & Snaydon, 1992; Lucas Bueno & Froud‐Williams, 1994). In ecological theory, uncertainty remains over the role of above‐ and belowground partitioning in determining the outcome of competition for nutrients (Aerts, 1999). Whilst it might be expected that a cultivar with vigorous shoot growth would also have a larger root system supported by the translocation of assimilate, it may also be the case that there is a trade‐off between above‐ and belowground partitioning. As techniques for measuring root growth develop, understanding of how root traits contribute to suppressive ability and tolerance in cereals is expected to improve.

Low input systems (such as organic agriculture) are more likely to suffer mineral deficiencies, and there could be more potential in developing cultivars that are more competitive for belowground resources. In an experiment comparing the relative importance of aboveground and belowground traits of *Arabidopsis thaliana* under different nutrient regimes, belowground traits contributed significantly to suppressive ability in nutrient‐poor conditions (Wang *et al*., 2010). The ability to forage for water is vital in many locations and may become increasingly important for determining the outcome of crop/weed competition in the context of predictions of widespread drought as a result of climate change (Stratonovitch *et al*., 2012). It has also been proposed that belowground traits determine the degree to which crop and weeds share resource pools (Smith *et al*., 2010), meaning more tolerant cultivars may be those with belowground traits which avoid resource pool overlap.

## The trade‐off between yield potential and weed competition

The majority of traits discussed above are not independent of one another and have implications for other plant functions in addition to weed competition, including yield potential and tolerance of stress. The higher potential yields of semidwarf modern cultivars have been achieved through changes in partitioning of carbon and nitrogen between stem and grain and more erect canopies with improved light use efficiency (Sinclair, 1998). But, as discussed above, shorter, more erect cultivars often have lower suppressive ability. Studies have established the link between suppressive ability and height, indicating that changes in allocation from the stem to the grain are at least partially responsible for the decreased competitive status of shorter varieties (Appleby *et al*., 1976; Challaiah *et al*., 1986; Siddique *et al*., 1989; Hoad *et al*., 2008). In low input systems, where weed pressure is often high, it may be acceptable to select cultivars on the basis of their competitive ability rather than their yield potential in weed‐free environments. However, for the majority of farmers, yield potential (as well as resistance to disease and lodging) will remain the primary criteria for the selection of cultivars and the main driver for breeders. Therefore, ideally, attention needs to be given to traits that confer greater competitive ability without incurring a yield reduction in the absence of weed competition. There is evidence that the trade‐off between weed competition and yield potential is not inevitable, suggesting that breeding for suppressive ability and weed‐free yield is a possibility in barley (Christensen, 1995; Didon & Bostrom, 2003; Bertholdsson, 2011) and in wheat (Reeves & Brooke, 1977; Coleman *et al*., 2001; Vandeleur & Gill, 2004). Huel and Hucl (1996) found that wheat cultivars with a high ability to withstand yield losses in weedy scenarios do not always rank low for weed‐free yield, implying that a trade‐off for tolerance need not necessarily be an impediment to breeding for competitive cultivars.

In addition, there may be trade‐offs between cultivar competitive ability and capability to resist stresses (Jordan, 1993). Such stresses could include limitation of resources other than light and space (such as water or minerals), resistance to crop pathogens and climatic extremes (Grime, 1977). Work in this area has been limited. Winter hardiness in winter barley had no bearing on competitive ability (Bertholdsson, 2011). However, work with *A. thaliana* suggests that lines with resistance to disease are less competitive in disease‐free scenarios than their susceptible counterparts (Damgaard & Jensen, 2002). Early vigour could be a penalty‐free competitive trait, but it has been suggested that, in drought conditions, this would result in rapid assimilation of all available soil moisture, leaving the resource limited during grain filling (Lemerle *et al*., 2001). Further work would be required to quantify these trade‐offs and identify win‐win traits that improve competitive ability without compromising other plant functions.

## Integrated management strategies

Growing a more competitive cultivar alone will not solve the problems associated with the more challenging conditions for weed control discussed in the introduction. Rather, their use will need to be integrated with a range of other cultural control strategies and the prudent use of herbicides. How cultivars interact with these other weed control options needs to be considered.

Delayed sowing has been shown to decrease infestations of certain weed species in winter wheat (Christensen *et al*., 1994; Cosser *et al*., 1997) and barley (Kolbe, 1980). This is primarily because a proportion of the autumn weed species germinate and may be controlled before the crop is sown, therefore fewer germinate within the crop. Variation in rate of development between cultivars means they may differ in their ability to maintain competitive ability at different sowing dates. A high rate of growth may also become more important when cereals are sown late (Hoad *et al*., 2006). Farmers are often unwilling to delay sowing because of the risk of missing the drilling window and an impact on yield, but these yield penalties will not be the same across cultivars. Knowledge of which cultivars can maintain a high yield when drilled later in the season will make this option more viable, especially in combination with suppressive ability. Faster developing cultivars may have a role to play in mitigating the risk to yield.

Increased sowing rate increases competitive ability, which has low cost of application compared with other control methods, and does not carry the same risks as delayed planting. It works on the principle that a greater mass of crop is present to compete against weeds, granting the population an asymmetric advantage to gather more resources and so further increase its advantage over weed populations (Weiner & Thomas, 1986). Increased seed rate has been observed to suppress weed growth in wheat (Christensen *et al*., 1994; Grundy *et al*., 1997; Champion *et al*., 1998; Roberts *et al*., 2001; Korres & Froud‐Williams, 2002) and barley (Paynter & Hills, 2009; Auskalniene *et al*., 2010). Mennan and Zandstra (2005) found that yield increased under higher sowing rates for wheat with and without competition from *G. aparine* and, at higher densities, greater suppression of the weed was achieved.

It is likely that there will be an interaction between cultivar choice and seed rate, in terms of weed suppression. However, some studies have found that sowing rate did not change the competitive rankings of cultivars in wheat (Cousens & Fletcher, 1990) and barley (O'Donovan *et al*., 2000), suggesting that it is a strategy highly compatible with using competitive cultivars. Other work has identified wheat cultivars that do not benefit from increased density (Korres & Froud‐Williams, 2002). This may indicate gaps in our understanding, although it could also mean that, in some circumstances, low‐density stands are able to intercept light as well as denser stands. Although some work suggests competitive ranking does not change with weed density (Cousens & Fletcher, 1990), other studies suggest that some traits, such as tillering ability, may vary in effectiveness depending on weed density that could explain differences between study results (Mason *et al*., 2008).

The interactions between cultivar traits and other cultural control options are complex and will also interact with weather (see below). Predicting the relative benefit of contrasting combinations of cultivar, sowing date and seed rate will require the application of mechanistic models of crop/weed competition (Kropff & Spitters, 1992; Deen *et al*., 2003; Storkey & Cussans, 2007).

## Screening for competitive ability

The idea of developing a ranking system for competitiveness of cultivars has been around for the past two decades. A screening protocol that predicts the competitive ability of cultivars would ideally be based on simple and rapid assessment of a selection of suitable traits (Cousens & Fletcher, 1990; Olesen *et al*., 2004). Such grading of cultivars should provide farmers with criteria to use when making management decisions (Lemerle *et al*., 2001). It would be more practical than current procedures, where cultivars would require many years of testing over a variety of conditions to get an accurate measure of suppressive ability (Brain *et al*., 1999). Farmers wishing to select a competitive cultivar often find that information on competitive ability is limited or based on subjective opinion.

Hansen *et al*. (2008) were successful in producing predictive ranking criteria for suppressive ability in spring barley. It was developed based on four traits – red/far‐red light reflectance at growth stage 31, leaf area index at growth stage 65, angle of leaves and the length of the culm, and was validated independently. However, some changes in ranking were observed in this study during independent assessments in differing locations, weakening the predictions of suppression. Including additional early growth traits has the potential to improve screening protocols, as they may have an important role in determining the competitive ability of cultivars, but they have received relatively little attention.

Competitive traits vary in their impact between years (Christensen, 1995; Huel & Hucl, 1996; Cosser *et al*., 1997; Cousens & Mokhtari, 1998; Coleman *et al*., 2001; Vandeleur & Gill, 2004; Bertholdsson, 2005), due to crop or weed species response to weather conditions. Variations across locations have resulted in lack of congruence in identifying traits responsible for competitive ability (Cousens & Mokhtari, 1998; Roberts *et al*., 2001). Not all studies report such variation. Suppressive abilities were consistent for barley cultivars in different years (Didon & Hansson, 2002). Lemerle *et al*. (1996) found that results were constant across years and sites, despite different weather patterns, and suggest that the similarity in soil type may have had a bearing on this. The strength of predictions could be increased through the use of multiple traits, as different traits may have greater importance under particular conditions (Christensen, 1995; Mennan & Zandstra, 2005).

## Conclusion: directions for future research

Many studies have illustrated the variation in competitive ability between cultivars. Traits that contribute to increased suppressive ability have been established, although the importance of these traits can vary between years. Further work would assist in clarifying the nature of the relationships between traits and competitive ability across sites and seasons. A rigorous screening protocol that ranks cultivars in a system similar to ranking for disease resistance would be most beneficial to farmers. Advice on how to best utilise competitive cultivars as part of an integrated weed management strategy would give growers more confidence in applying this approach.
